# Editorial: Inner ear therapeutics: the road to hearing rehabilitation and restoration

**DOI:** 10.3389/fnins.2024.1477915

**Published:** 2024-09-30

**Authors:** Sandra Prentiss, Athanasia Warnecke

**Affiliations:** ^1^Leonard M. Miller School of Medicine, University of Miami, Miami, FL, United States; ^2^Department of Otorhinolaryngology, Hannover Medical School, Hanover, Germany

**Keywords:** regenerative medicine, therapeutic hypothermia, inner ear therapeutics, intracochlear drug delivery, cochlear health

In recent years, advancements in inner ear therapeutics have brought us closer to the dream of effective hearing rehabilitation and restoration. The collection of articles in this Research Topic represents a significant stride toward understanding the complexities of inner ear disorders and developing innovative therapies to address them. This editorial aims to frame the central themes and objectives of the research presented, highlighting the progress made and the future directions in this vital field.

## Bridging the gap: from understanding to intervention

Hearing loss affects millions worldwide, impacting communication, social interaction, and quality of life. Traditional hearing aids and cochlear implants provide some relief but do not fully restore natural hearing. The research in this Research Topic emphasizes the importance of a multidisciplinary approach, integrating molecular biology, genetics, pharmacology, and bioengineering to develop more effective treatments.

One key study by Grzybowski et al. entitled, “*Optimization of pharmacological interventions in the guinea pig animal model*,” explores a novel method to calculate the perilymph volume of the scala tympani (ST) in guinea pigs. This approach considers individual differences in the size of the guinea pig cochlea, a factor previously overlooked, to optimize drug concentrations and improve pharmacotherapy outcomes. High-resolution μCT images were used to measure ST volume in various tissue conditions and strains, demonstrating variability similar to human cochleae and underscoring the importance of accounting for this variability in research. Improved calculation of perilymph volume would allow for more precise dosing of medications for inner ear therapy. This could enhance the efficacy and safety of clinical trials, potentially leading to more effective treatments for inner ear disorders in humans.

## Regenerative medicine: a new frontier

Regenerative medicine holds the potential to restore hearing by repairing or replacing damaged cells in the inner ear. Stem cell therapy and tissue engineering are at the forefront of this research. The study by Schwieger et al.: “*Of Mice and Men: The relevance of Cometin and Erythropoietin origin for its effects on murine spiral ganglion neuron survival and neurite outgrowth in vitro*” investigates the neuroprotective potential of neurotrophic factors (NTFs) like Cometin and Erythropoietin (EPO) on spiral ganglion neurons (SGN). The findings indicate that human-derived Cometin significantly enhances SGN survival and neurite outgrowth, unlike its murine counterpart, highlighting the importance of species origin in therapeutic screenings. The finding that human-derived Cometin promotes survival and growth of spiral ganglion neurons opens new possibilities for therapies aimed at preserving and regenerating auditory nerve cells. This could lead to improved treatment options for patients with hearing loss.

## Therapeutic hypothermia: a promising avenue

Two studies in this Research Topic explore the potential of therapeutic hypothermia for mitigating noise-induced hearing loss (NIHL). The study “*Transcriptional response to mild therapeutic hypothermia in noise-induced cochlear injury*” by Rincon Sabatino, Sangaletti et al. shows that localized hypothermia post-noise exposure mitigates acute cochlear injury by reducing inflammatory responses. Similarly, another promising approach discussed in this Research Topic is detailed in the study by Rincon Sabatino, Rivero et al., titled “*Targeted Therapeutic Hypothermia Protects Against Noise-Induced Hearing Loss*”. The researchers developed a custom-designed cooling neck collar that reduces the temperature of the inner ear by 3–4°C following noise exposure, providing localized and non-invasive therapeutic hypothermia. This method successfully mitigated noise-induced hearing loss (NIHL) in rats, preserving residual hearing and rescuing noise-induced synaptopathy over an extended period. This innovative approach has a high potential for rapid clinical translation, offering a new avenue for the long-term preservation of hearing health. The development of a non-invasive method for local cooling of the inner ear offers a promising approach to prevent noise-induced hearing loss. This method has high potential for rapid clinical translation and could serve as an effective preventive measure for individuals exposed to high noise levels.

## Clinical applications and future directions

While preclinical research is vital, translating these findings into clinical applications is the ultimate goal. The study “*Deep intracochlear injection of triamcinolone-acetonide with an inner ear catheter in patients with residual hearing*” by Prenzler et al. investigates the safety and efficacy of intracochlear steroid application in cochlear implant recipients. The results indicate that this method does not significantly impact residual hearing, suggesting it could be a feasible approach for drug delivery in clinical settings. Thus, direct administration of steroids into the inner ear seems to be safe for cochlear implant recipients. This opens up new possibilities for targeted drug delivery in the inner ear and could improve outcomes of cochlear implantations.

Despite significant progress, systemic barriers remain in the development of inner ear therapeutics, as discussed in the review by Jiam and Rauch “*Inner Ear Therapeutics and the War on Hearing Loss: Systemic Barriers to Success*.” This article highlights the need for improved diagnostics, increased collaboration, and a robust drug development infrastructure to overcome these challenges and achieve FDA approval for inner ear treatments. Identifying obstacles in the development of inner ear therapeutics helps in developing strategies to overcome barriers, to pave the way for FDA-approved treatments and to ultimately lead to wider availability of effective therapies for patients with hearing loss.

## Conclusion

The research presented in this Research Topic represents a significant step forward in our understanding and treatment of hearing loss. By addressing the fundamental biological mechanisms, developing innovative delivery systems, and exploring regenerative therapies, these studies offer hope for effective hearing rehabilitation and restoration. The journey is far from over, but the progress made thus far is a testament to the dedication and ingenuity of researchers in this field.

We extend our gratitude to all contributors for their valuable insights and groundbreaking work. Together, we are paving the way toward a future where hearing loss can be effectively managed and even reversed, improving the lives of millions around the world.

